# A paraneoplastic limbic encephalitis from an anorectal small cell neuroendocrine carcinoma: a case report

**DOI:** 10.1186/s12883-019-1542-9

**Published:** 2019-11-29

**Authors:** Raffaele Longo, Marc Wagner, Benjamin Savenkoff, Mathilde Chastenet de Castaing, Guillaume Desiro, Zead Tubail, Laurent Hennequin, Sinan Ben Mahmoud, Nathalie Marcon, Philippe Quetin, Marco Campitiello, Francesca Plastino

**Affiliations:** 1 Division of Medical Oncology, “CHR Metz-Thionville”, 1 Allée du Château, 57085 Ars-Laquenexy, France; 2 Division of Neurology, “CHR Metz-Thionville”, 1 Allée du Château, 57085 Ars-Laquenexy, France; 3 Division of Nephrology, “CHR Metz-Thionville”, 1 Allée du Château, 57085 Ars-Laquenexy, France; 4 Division of Radiology, “CHR Metz-Thionville”, 1 Allée du Château, 57085 Ars-Laquenexy, France; 5 Division of Nuclear Medecine, “CHR Metz-Thionville”, 1 Allée du Château, 57085 Ars-Laquenexy, France; 6 Division of Pathology, “CHR Metz-Thionville”, 1 Allée du Château, 57085 Ars-Laquenexy, France; 7 Division of Radiotherapy, “CHR Metz-Thionville”, 1 Allée du Château, 57085 Ars-Laquenexy, France

**Keywords:** Limbic encephalitis, Paraneoplastic, Small cell carcinoma

## Abstract

**Background:**

Paraneoplastic limbic encephalitis (PLE) is a rare autoimmune neurological syndrome observed in cancer patients. PLE is difficult to diagnose and presents a variable response to treatment, depending on the characteristics of the tumor and neuronal autoantibodies.

**Case presentation:**

A 64-year-old, Caucasian, non-smoker man presented with a rapidly developing cognitive impairment, personality change, spatial disorientation, and short-term memory loss associated with anorexia and cervical and inguinal lymph nodes. The ^18^F-FDG PET scan documented intensely hypermetabolic lymph nodes, which histologically corresponded to a metastasis from a small cell neuroendocrine carcinoma. The brain MRI revealed a high T2-weighted FLAIR signal of the hippocamps, consisted with a PLE. The presence of anti-neuronal Hu antibodies confirmed the diagnosis. The patient underwent plasmapheresis, associated to a systemic chemotherapy resulting in a partial and temporary improvement of the neurological symptoms. Four cycles of intravenous immunoglobulins were also necessary. After six cures of chemotherapy, the lymph node metastases regressed. However, a new anorectal lesion was detected and was histologically confirmed as a primary small cell neuroendocrine carcinoma, which was treated with concomitant chemoradiotherapy. At the end of this treatment, the patient showed a rapid tumor progression leading to his death.

**Conclusions:**

This case highlights the rare entity, PLE, which is difficult to diagnose and manage. In addition, this is the first published case of PLE associated with an anorectal small cell neuroendocrine carcinoma, which appeared after completion of systemic chemotherapy.

## Background

Paraneoplastic limbic encephalitis (PLE) is an uncommon autoimmune neurological syndrome observed in cancer patients selectively affecting the limbic areas, including hyppocampus, amygdala, hypothalamus, cingulate gyrus and limbic cortex [1–6]. PLE is clinically characterized by subacute neuropsychiatric symptoms, such as altered mental status, dementia, mood changes, short-term memory deficit, confusion and seizures [3–6]. At the magnetic resonance imaging (MRI), PLE shows a typical increased signal on T2-weighted FLAIR imaging in the medial temporal lobes [3–6]. The diagnosis must be supported by the identification of specific neuronal autoantibodies [6]. The management is complex and based on a multidisciplinary and combined approach of immunotherapy and specific anticancer treatment [7–26]. The prognosis is usually poor and strictly related to the subtype of neuronal autoantibodies, clinical patient’s status and comorbidities, and cancer characteristics and stage [24–26].

We report a case of a patient showing a PLE associated to a rare, metastatic, anorectal small cell neuroendocrine carcinoma. The primary tumor was diagnosed after completion of systemic chemotherapy.

## Case presentation

**Fig. 1:**
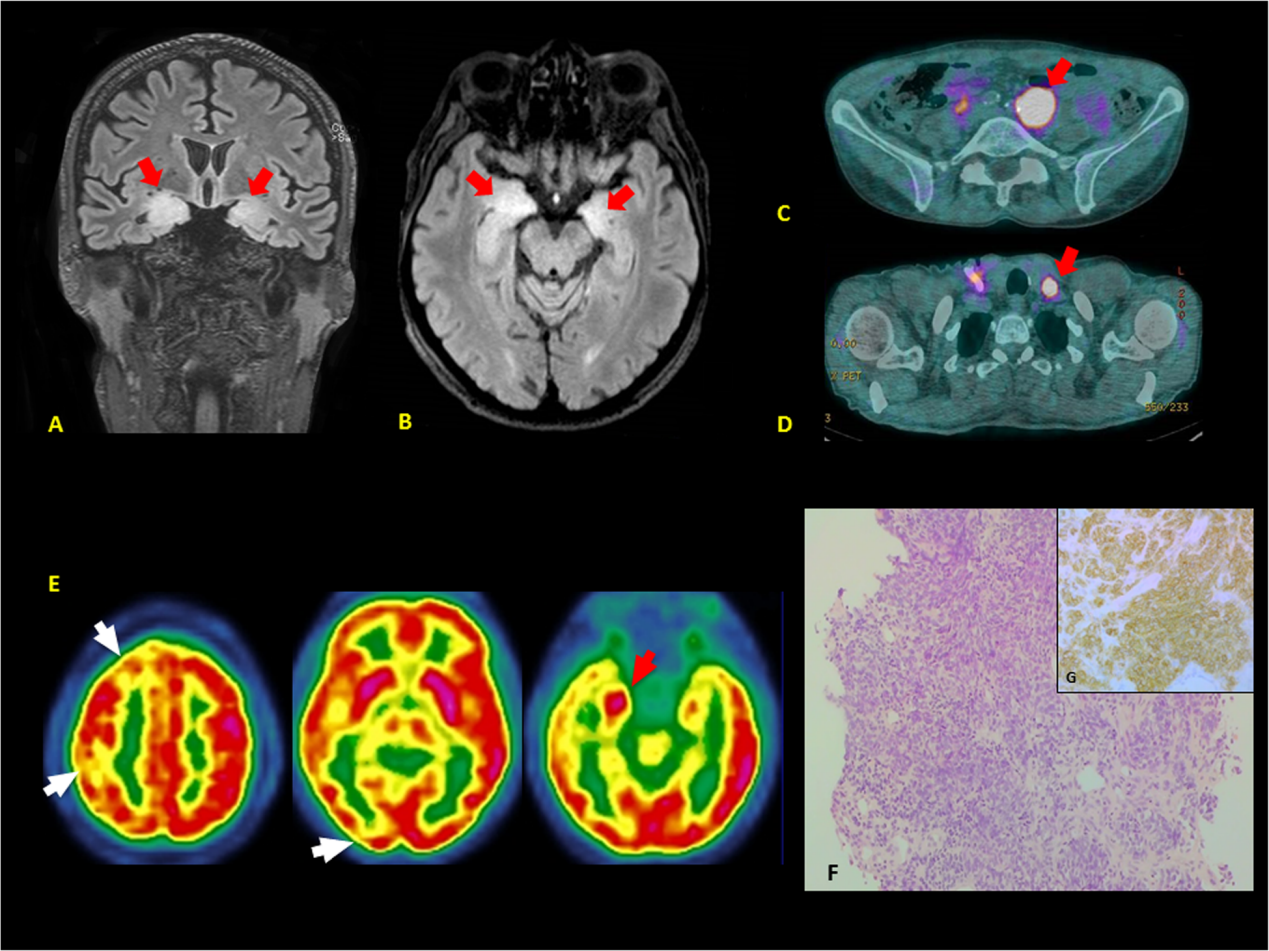
Diagnosis of PLE associated with lymph node metastases of a small cell neuroendocrine carcinoma. **a-b** T2-weighted FLAIR signal of the para hippocampic gyrus and the hippocamps (brain MRI: coronal and axial section; red arrows). **c-d** Left, hypermetabolic peri-aortic and supraclavicular lymph node (PET-scan; red arrows). **e** Right hypermetabolism of the hippocampus (PET-scan; red arrow) and a right prefrontal, parietal and occipital hypometabolism (white arrows). **f** Massive tumor infiltration of poorly differentiated cells with scant cytoplasm, coarse chromatin, prominent mitotic and apoptotic figures, associated to a diffuse necrosis (histology; hematoxylin and eosin stain, 200x). **g** Positivity of tumor cells for CD56 (immunohistochemistry; 400x)

A 64-year-old, Caucasian, non-smoker man was admitted to the neurology division for acute cognitive impairment, personality change, spatial disorientation, and short-term memory loss associated with anorexia. He had no relevant comorbidities. The patient’s history was uneventful. At the clinical examination, we found hard, irregular, cervical and inguinal lymph nodes of 1.5 × 1.5 cm of diameter. Biological tests were all in the normal ranges but chromogranin-A and neuron-specific enolase (NSE) were elevated at 175 ng/ml (NV < 102 ng/ml) and 28 ng/ml (NV < 17 ng/ml). The brain MRI revealed T2-weighted fluid-attenuated inversion recovery (FLAIR) signals of the para hippocampic gyrus and the hippocamps (Fig. 1a-b**, red arrows**). The electroencephalogram (EEG) showed a 1-min, left, fronto-temporal focal crisis. The ^18^F-FDG (^18^F-fluorodeoxyglucose) PET scan documented many cervical and inguinal hypermetabolic lymph nodes (Fig. 1c-d**, red arrows**), a hypermetabolism of the posterior surface of the right prostate lobe and the right hippocampus (Fig. 1e**, red arrow**), and a right prefrontal, parietal, and occipital cortical hypometabolism (Fig. 1e**, white arrows**).

The patient referred to a percutaneous ultrasound-guided core biopsy of both, cervical and inguinal, lymphadenopathies and the prostate gland. Histology revealed a massive infiltration of small, poorly differentiated, tumor cells with a scant cytoplasm and prominent mitotic figures (Fig. 1f). At the immunohistochemistry, tumor cells were positive for CD56 (Fig. 1g) and synaptophysin, and negative for cytokeratin AE1/AE3, CK-7, CK-20, chromogranin-A, p53, PAX8, GATA-3, and TTF-1 according to the diagnosis of a lymph node metastasis of a poorly differentiated, small cell neuroendocrine tumor. The proliferation index (Ki-67) was elevated at 90%. Prostate histology was consisted with a prostatitis. The lumbar ponction revealed a clear pleocytose (1 white cell/mm^3^; NV < 1 cells/mm^3^) and elevated protein levels (1,15 g/L; NV < 0,40 g/L) without any evidence of tumor cells. We documented the presence of serum anti-neuronal Hu antibodies in the absence of other neuronal autoantibodies, including anti-Yo, anti-Ri, anti-CV2, anti-Ma2, anti-SOX1, anti-GAD65, anti-Tr, anti-Zic4, anti-Titin, anti-amphiphysin, anti leucine-rich glioma inactivated 1 (LGl1), and anti contactin associated protein 2 (CASPR2). The molecular analysis of Cytomegalovirus and Human Herpesvirus-6 (HHV-6) genome in the cerebrospinal fluid (CSF) was negative. This biological pattern confirmed the diagnosis of PLE.

**Fig. 2:**
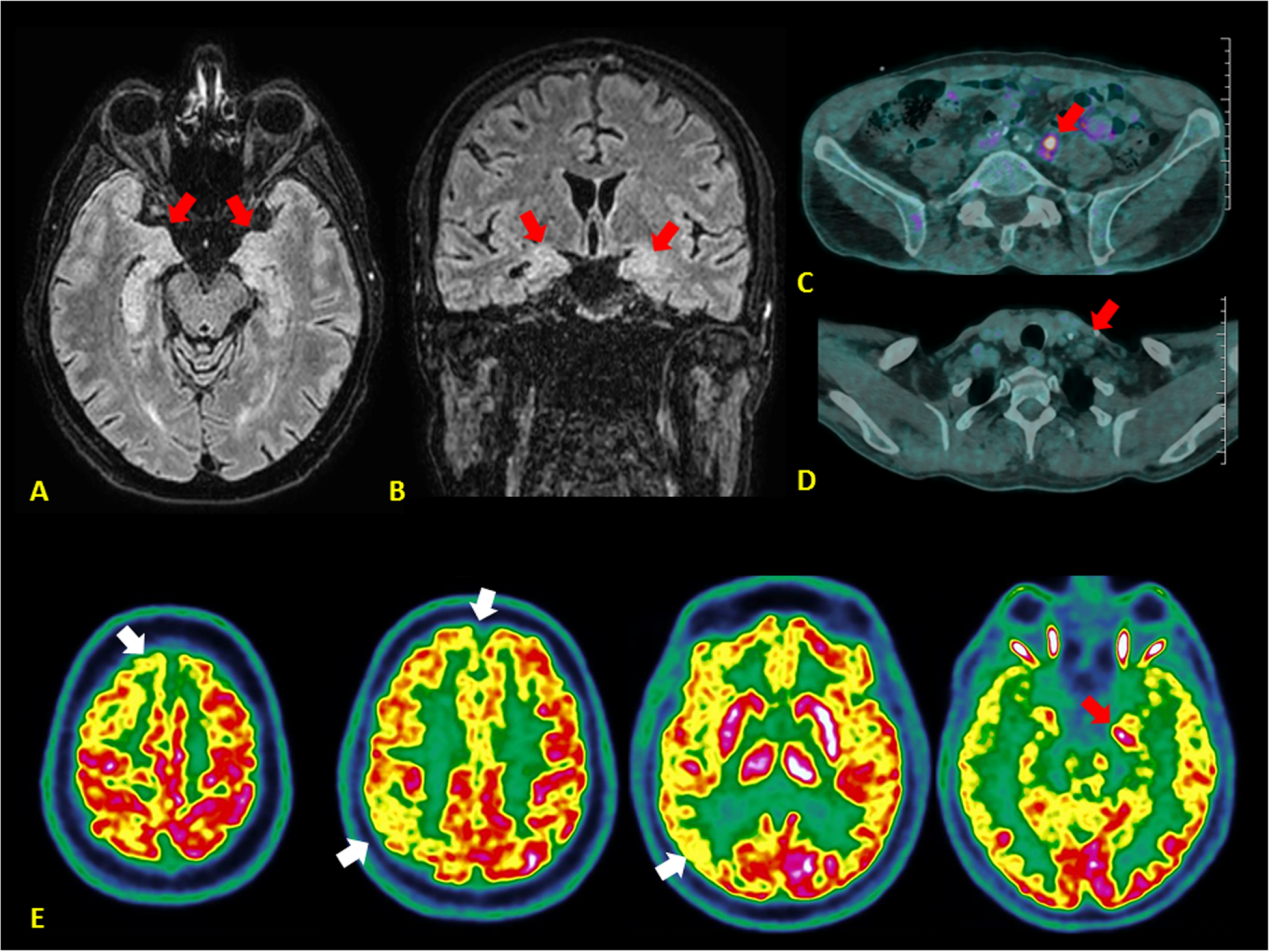
Tumor response to chemotherapy and radiological improvement of the PLE. **a-b** Reduction of the T2-weighted FLAIR signal of the para hippocampic gyrus and the hippocamps after 3 cycles of chemotherapy (brain MRI: coronal and axial section; red arrows). **c-d** Partial regression of the left, hypermetabolic peri-aortic adenopathy and complete response of the left, hypermetabolic supraclavicular lymph node after 6 cycles of chemotherapy (PET-scan; red arrows). **e** Residual, left hypermetabolism of the hippocampus (brain PET-scan; red arrow) and right prefrontal, parietal and occipital cortical hypometabolism (brain PET-scan; white arrows) after 6 cycles of chemotherapy

The patient underwent several cycles of plasmapheresis combined with high-dose corticosteroids that partially and temporarily improved neurological symptoms. We started also a systemic chemotherapy by a cisplatin/etoposide regimen. After three cycles of chemotherapy, lymph node metastases partially regressed. The brain MRI revealed a radiological improvement of the limbic lesions (Fig. 2a-b**, red arrows**) but patient’s neurological symptoms quickly worsened and the patient presented with many episodes of partial epileptic seizures and confusion. In this context, we administered four cycles of intravenous immunoglobulins (IVIgs) in addition to the systemic chemotherapy leading to a rapid and progressive improvement of the neurological symptoms.

**Fig. 3:**
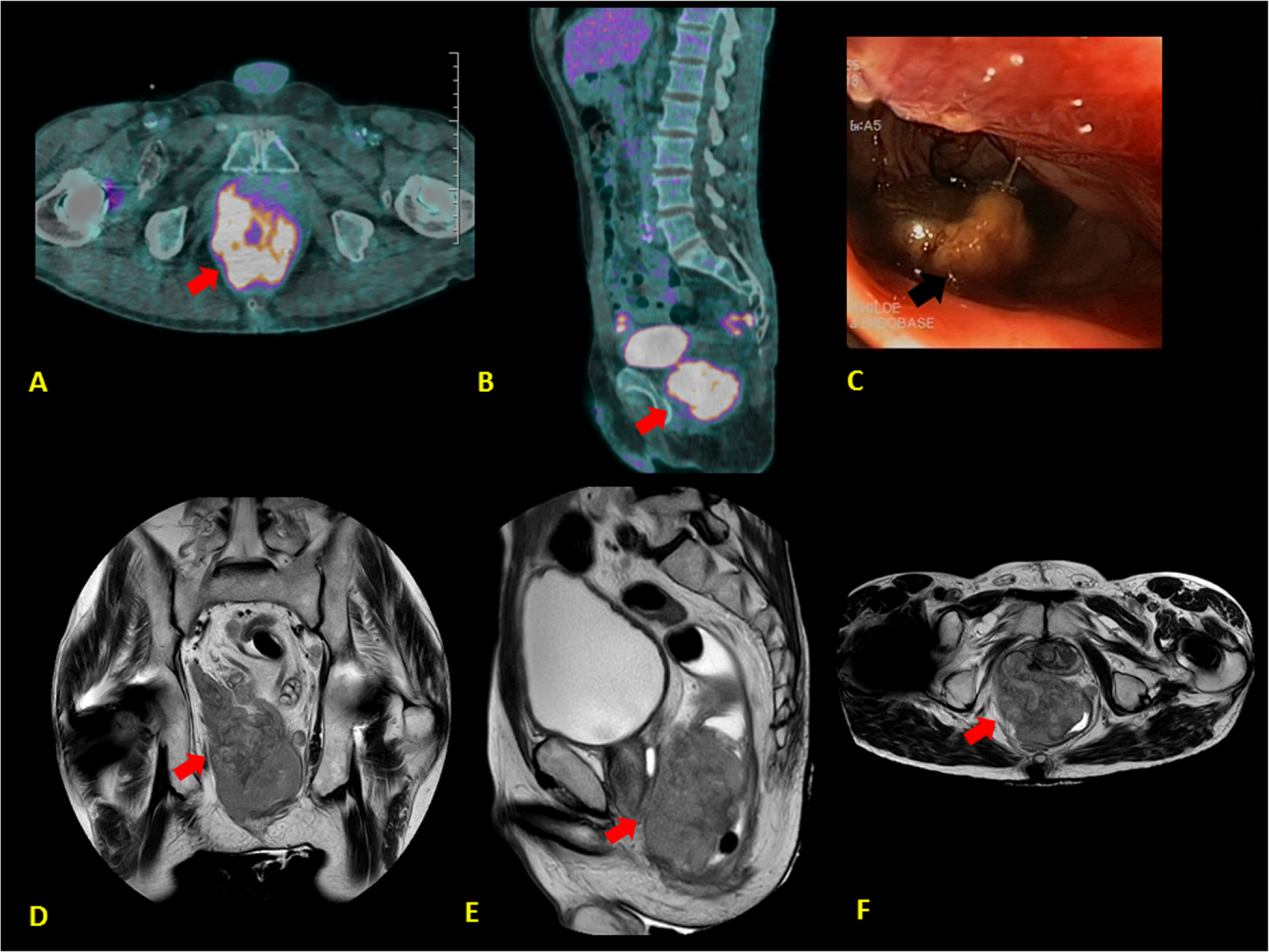
Diagnosis of a primary, anorectal small cell tumor. **a-b** Voluminous, hypermetabolic anorectal tumor lesion (PET-scan: axial and sagittal section; red arrows) diagnosed after 6 cycles of chemotherapy. **c** Tumor lesion of the right wall of the anorectal region (Colonoscopy; black arrows**)**. **d**-**f** Voluminous tumor of the right rectal wall of 62 × 84 × 162 mm of diameter, infiltrating the prostate, the right seminal vesicle, the internal anal sphincter, the levator ani and the puborectalis muscle (pelvic MRI: coronal, sagittal and axial section; red arrows)

After six cycles of systemic chemotherapy, the whole body CT and ^18^F-FDG PET scan showed a regression of metastatic lymphadenopathies (Fig. 2c-d**, red arrows**), an improvement of brain abnormalities (Fig. 2e**, red and white arrows**) but the presence of a new, anorectal, hypermetabolic tumor lesion (Fig. 3a-b**, red arrows**). The colonoscopy (Fig. 3c**, black arrow**) and pelvic MRI (Fig. 3d-e**, red arrows**) documented a voluminous tumor of the right rectal wall of 62 × 84 × 162 mm of diameter, infiltrating the prostate, the right seminal vesicle, the internal anal sphincter, the levator ani and the puborectalis muscle. The percutaneous biopsy revealed a small cell neuroendocrine carcinoma.

In order to obtain a better control of this new tumor lesion, we started a concomitant chemoradiotherapy with a carboplatin/etoposide regimen. After completion of treatment, the patient developed a diplopia, which was related to a left intraorbital metastasis. The ^18^F-FDG PET scan showed also lung, hepatic, lymph node, and peritoneal multiple metastases. In this contexte, we administered a palliative systemic chemotherapy by weekly topotecan but the patient’s clinical conditions quickly worsened leading to his death.

## Discussion and conclusion

PLE is a rare, autoimmune neurological syndrome affecting the limbic areas [1–11]. It is usually associated with small cell lung cancer, breast cancer, thymoma, ovarian teratoma and Hodgkin lymphoma [1–11].

PLE is clinically characterized by subacute confusion, altered mental status, mood changes, short-term memory deficit, dementia and seizures [1–5]. The subacute development of short-term memory loss (< 3 months) is usually considered the hallmark of PLE, but other neurological symptoms, such as cerebellar ataxia, progressive encephalomyelopathy, and peripheral neuropathy may be more clinically relevant [3, 4].

CSF analysis shows a mild-to-moderate lymphocytic pleocytosis (usually < 100 white blood cells/mm^3^) in 60–80% of patients and an elevated IgG index or oligoclonal bands in approximately 50% of cases [3–6].

Brain MRI often reveals an increased signal on T2-weighted FLAIR imaging in the medial temporal lobes, the unilateral or the absence of the temporal involvement being very uncommon [3–6]. In this latter case, the diagnosis should be confirmed by the identification of specific anti-neuronal antibodies as several non-immune, neurological disorders could present a similar unilateral radiological picture, including seizures, HHV-6 encephalitis or gliomas [3–6]. HHV-6 encephalitis can precisely mimic PLE but it often shows a particular radiological pattern, including a less confined involvement of the limbic system, haemorrhagic aspects, restricted diffusion abnormalities and contrast uptake, and prominent signs of oedema and mass effect involving one or both inferior-medial temporal lobes, inferior frontal lobes and cingulate gyrus. In addition, the clinical setting is different [3–6].

Several published studies have clearly confirmed the pathogenic role of few anti-neuronal antibodies in PLE. Classical PLE with temporal lobe seizures is associated with onconeural autoantibodies directed against intracellular antigens, including anti-Hu, anti-Ma2, anti-amphiphysin, and anti collapsin response mediator protein 5 (CRMP5). Recently, many autoantibodies to neuronal extracellular epitopes have been described, such as the voltage-gated potassium channel (VGKC) complex, N-methyl-D-aspartate receptor (NMDAR), α-amino-3-hydroxy-5-methyl-4-isoxazolepropionic acid receptor (AMPAR), γ-aminobutyric acid B receptor (GABA_B_R), LGI1, and CASPR2 [3–6, 12].

Histologically, PLE shows particular inflammatory features, including perivascular lymphocytic cuffs, neuronal loss, reactive microglial nodules and gliosis of the temporal lobe [3].

The diagnosis remains a challenge and generally requires a combination of clinical, biological, and radiological findings [3–6]. Additionally, metabolic and inflammatory encephalopathy, neurotoxic drugs, brain tumors and neurodegenerative disorders must be excluded [3–6]. The brain MRI is more useful to rule out other disorders [3]. The ^18^F-FDG PET scan might be necessary, particularly when MRI is not conclusive [13–22].

The identification of specific circulating autoantibodies is essential for a definitive diagnosis [3–6]. The onconeural antibodies targeting intracellular proteins of the neuroectodermal tissues are usually characterized by a permanent lymphocyte-T mediated neuronal loss with a low response to the immunomodulatory treatment. On the contrary, the prognosis of PLE associated to the autoantibodies targeting the cell surface antigens is usually better as the neuronal dysfunction is often reversible after the immunosuppressive treatment [3–6].

The management of PLE is complex and based on a multidisciplinary combined approach, including immunotherapy and specific anticancer treatment [23–26]. Different immunomodulator treatments, such as corticosteroids, plasma exchange, immune adsorption or IVIgs, have been reported in the literature with variable results [23–26]. The anticancer treatment plays a pivotal role and should always be used in combination with immunotherapy as this combined approach clearly appears more effective [23–26]. A relatively small percentage of PLE patients experienced symptom stabilization or neurological improvement after immunomodulatory therapy, although most failed to achieve a response until the primary tumor was controlled [24, 25].

In our case, the patient presented with a PLE associated to a rare anorectal small cell neuroendocrine carcinoma that was diagnosed after completion of systemic chemotherapy. Considering the lack of response to plasmaferesis and high-dose corticosteroids, four cycles of IVIgs were administered in addition to chemotherapy leading to a quickly clinical improvement of neurological symptoms. We performed also a concomitant chemoradiotherapy for a better control of the anorectal tumor but the patient showed a rapid, multi-visceral, tumor progression leading to his death.

This case highlights the rare entity, PLE, which is difficult to diagnose and manage. At our knowledge, it is the first published case of a PLE associated to an anorectal small cell neuroendocrine carcinoma. The diagnosis of the primary tumor was a challenge as the patient presented only with metastatic lymph nodes at his first clinical examination. The ^18^F-FDG PET scan showed an abnormal hypermetabolism of the posterior surface of the right prostate lobe. The clinical examination and the prostate biopsy did not document any pathological lesion but this does not exclude a microscopic tumor of the anorectal mucosa. It is impossible to histologically discriminate a primary anorectal tumor from a metastasis. However, despite the excellent response of lymph node metastases after completion of chemotherapy, a new, voluminous anorectal tumor was diagnosed at the same site where the first ^18^F-FDG PET scan found the abnormal hypermetabolism. All these considerations and the clinical behavior of this new lesion, which progressed during chemotherapy in contrast to lymph node metastases, support the hypothesis of a primary anorectal tumor, which was resistant to chemotherapy.

## Data Availability

The datasets used and/or analysed during the current study are available from the corresponding author on raisonable request. All data and materials are available for review at the Division of Medical Oncology, CHR Metz-Thionville, in an electronic format.

## Abbreviations

^18^F-FDG: ^18^F-fluorodeoxyglucose
AMPAR: α-amino-3-hydroxy-5-methyl-4-isoxazolepropionic acid receptor
CRMP5: Collapsin response mediator protein 5
CSF: Cerebrospinal fluid
EEG: Electroencephalogram
FLAIR: Fluid-attenuated inversion recovery
GABA_B_R: γ-aminobutyric acid B receptor
HHV-6: Human Herpesvirus-6
IVIgs: Intravenous immunoglobulins
LE: Limbic encephalitis
MRI: Magnetic resonance imaging
NMDAR: N-methyl-D-aspartate receptor
NSE: Neuron-specific enolase
PLE: Paraneoplastic limbic encephalitis
VGKC: Voltage-gated potassium channel
