# Correction to: A paraneoplastic limbic encephalitis from an anorectal small cell neuroendocrine carcinoma: a case report

**DOI:** 10.1186/s12883-022-02598-1

**Published:** 2022-03-08

**Authors:** Raffaele Longo, Marc Wagner, Benjamin Savenkoff, Mathilde Chastenet de Castaing, Guillaume Desiro, Zead Tubail, Laurent Hennequin, Sinan Ben Mahmoud, Nathalie Marcon, Philippe Quetin, Marco Campitiello, Francesca Plastino

**Affiliations:** 1 Division of Medical Oncology, “CHR Metz-Thionville”, 1 Allée du Château, 57085 Ars-Laquenexy, France; 2 Division of Neurology, “CHR Metz-Thionville”, 1 Allée du Château, 57085 Ars-Laquenexy, France; 3 Division of Nephrology, “CHR Metz-Thionville”, 1 Allée du Château, 57085 Ars-Laquenexy, France; 4 Division of Radiology, “CHR Metz-Thionville”, 1 Allée du Château, 57085 Ars-Laquenexy, France; 5 Division of Nuclear Medecine, “CHR Metz-Thionville”, 1 Allée du Château, 57085 Ars-Laquenexy, France; 6 Division of Pathology, “CHR Metz-Thionville”, 1 Allée du Château, 57085 Ars-Laquenexy, France; 7 Division of Radiotherapy, “CHR Metz-Thionville”, 1 Allée du Château, 57085 Ars-Laquenexy, France

**Correction to: BMC Neurol 19, 304 (2019)**

**https://doi.org/10.1186/s12883-019-1542-9**

Following publication of the original article [1], the authors would like to correct the statement under Case presentation, second paragraph.

The sentence currently reads:

“The lumbar ponction revealed a clear pleocytose (1 white cell/mm3; NV < 1 cells/mm3) and elevated protein levels (1,15 g/L; NV < 0,40 g/L) without any evidence of tumor cells. We documented the presence of serum anti-neuronal Hu antibodies…”

The sentence should read:

“The lumbar ponction **did not reveal a pleocytosis** (1 white cell/mm3; NV < 1 cells/mm3) **but** elevated protein levels (1,15 g/L; NV < 0,40 g/L) without any evidence of tumor cells. We documented the presence in the serum and** in the cerebrospinal fluid (CSF) of** anti-neuronal Hu antibodies…”

The original article [1] has been updated.
